# Research Progress on Quantum Dot-Embedded Polymer Films and Plates for LCD Backlight Display

**DOI:** 10.3390/polym17020233

**Published:** 2025-01-17

**Authors:** Bin Xu, Jiankang Zhou, Chengran Zhang, Yunfu Chang, Zhengtao Deng

**Affiliations:** 1 Department of Electronic Information Engineering, School of Computer and Information Engineering, Nanjing Tech University, Nanjing 211816, China; changyunfu@njtech.edu.cn; 2 College of Engineering and Applied Sciences, Nanjing University, Nanjing 210023, China; 522023340080@smail.nju.edu.cn (J.Z.); zhangchengran@ntchanged.com (C.Z.)

**Keywords:** quantum dot, polymers, backlight display, display technology

## Abstract

**Abstract:** Quantum dot–polymer composites have the advantages of high luminescent quantum yield (PLQY), narrow emission half-peak full width (FWHM), and tunable emission spectra, and have broad application prospects in display and lighting fields. Research on quantum dots embedded in polymer films and plates has made great progress in both synthesis technology and optical properties. However, due to the shortcomings of quantum dots, such as cadmium selenide (CdSe), indium phosphide (InP), lead halide perovskite (LHP), poor water, oxygen, and light stability, and incapacity for large-scale synthesis, their practical application is still restricted. Various polymers, such as methyl methacrylate (PMMA), polyethylene terephthalate (PET), polystyrene (PS), polyvinylidene fluoride (PVDF), polypropylene (PP), etc., are widely used in packaging quantum dot materials because of their high plasticity, simple curing, high chemical stability, and good compatibility with quantum dot materials. This paper focuses on the application and development of quantum dot–polymer materials in the field of backlight displays, summarizes and expounds the synthesis strategies, advantages, and disadvantages of different quantum dot–polymer materials, provides inspiration for the optimization of quantum dot–polymer materials, and promotes their application in the field of wide-color-gamut backlight display.

## 1. Introduction

**Figure 1:**
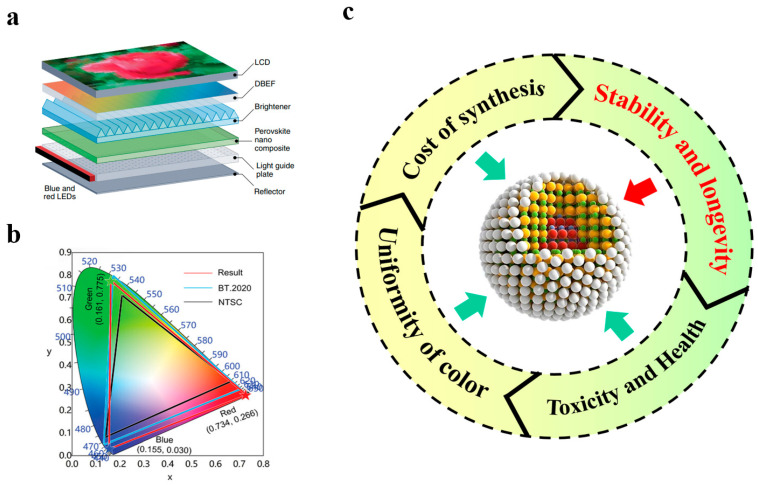
(**a**) Photoluminescent liquid crystal display structure schematic diagram. Reprinted with permission from ref. [1]. Copyright 2020 Springer Nature. (**b**) Gamut in CIE chromaticity diagram. Reprinted with permission from ref. [4]. Copyright 2023 Wiley. (**c**) Current problems and challenges faced by quantum dot materials.

With the continuous progress of semiconductor technology, display technology has witnessed remarkable enhancements over the last several decades, particularly in aspects such as color gamut width, luminance, and lifespan. The backlight display technology currently employed in the market is mainly the photoluminescence (PL) type (Figure 1a). Photoluminescence technology is commonly seen in liquid crystal displays (LCD), where blue light LEDs and down-converting luminescent materials (such as phosphor or quantum dots) are utilized as the backlight source [1]. Among the multiple components of the display device, the luminescent material is the core factor determining the display performance. To offer superior display quality, the luminescent material must possess several key characteristics: high luminous efficiency—the material is capable of effectively converting input energy into light, thereby enhancing display luminance; high color purity—the light emitted by the material needs to have a distinct color to ensure clear and accurate displayed colors; and good stability—the material should maintain stable performance during long-term usage or under high-temperature conditions, preventing color distortion or luminance attenuation. These characteristics jointly determine the visual performance and service life of the display device [2,3]. Hence, the selection and optimization of luminescent materials are crucial for improving the quality of display technology.

Currently, most of the LCD backlight display materials available in the market employ blue light LED chips combined with a coating of yellow fluorescent materials (such as YAG:Ce^3+^) to generate white light. Nevertheless, this approach confronts significant issues. Since the spectrum produced by blue light LED and yellow phosphor does not fully encompass the entire visible spectrum, particularly in the red and green regions, it leads to inaccurate color reproduction and low saturation [5]. The color gamut of this backlighting scheme is typically narrow, merely approximately ~72% of the National Television System Committee (NTSC) standard (Figure 1b), making it challenging to achieve higher standards of color performance. The light generated by the strategy of blue light LED and yellow fluorescent materials usually leans towards a cool hue (high color temperature), and thus the overall display effect might appear cold. To compensate for this, it is often necessary to adjust the color temperature of the backlight, but excessive adjustment might result in the distortion of other colors. The LCD panel itself has a certain degree of viewing angle dependence. When using a blue light LED backlight, the performance of color and luminance may vary at different viewing angles, especially when viewed at larger angles, and the problem of uneven color and luminance might be more pronounced. The spectrum of blue light LED contains a relatively larger proportion of short-wavelength blue light. Prolonged viewing might cause certain levels of eye fatigue, and some studies have even raised concerns about potential adverse effects on sleep quality, especially following excessive exposure to a strong blue light environment [6,7,8,9,10,11].

Quantum dots, as luminescent materials, can significantly enhance the color gamut and luminance of LED-backlit LCD displays with their outstanding color performance and high efficiency, thereby delivering more vivid and precise visual experiences. The application of this technology enables liquid crystal display televisions to present richer and more brilliant colors, particularly in the display of high dynamic range (HDR) content [12,13,14,15,16].

In 2013, Sony pioneered the launch of the world’s first liquid crystal television (QD-LCD) equipped with quantum dot backlight technology, signifying the official advent of the quantum dot television era. In 2014, TCL showcased quantum dot LCD televisions at the International Consumer Electronics Show (CES) and the IFA exhibition in Berlin. This innovative technology promptly aroused extensive attention within the industry. Subsequently, renowned domestic and international television brands such as Samsung followed suit, and quantum dot televisions rapidly emerged as a significant new category in the television industry, highly favored by consumers [17].

However, the application of quantum dot materials in high-performance display devices still presents numerous issues that urgently require resolution (Figure 1c). Firstly, the problem of water and oxygen stability of quantum dots must be addressed [18,19,20,21]. Therefore, this article focuses on the research progress on quantum dot polymers in the field of backlight display, analyzes the important indicators of backlight display, contrasts the pros and cons of different types of quantum dots, conducts a detailed discussion on various encapsulation methods of quantum dots and polymers, and finally puts forward some challenges and opportunities in this domain, facilitating the development of the quantum dot backlight display field.

## 2. Important Indicators for Backlight Display and Related Parameters of Quantum Dots

### 2.1. Color Gamut of Backlight Display and FWHM

Color gamut refers to the range of colors that display devices (such as televisions, computer monitors, printers, etc.) are capable of displaying or reproducing. Different devices have varying color gamut sizes, and thus the types and saturation of colors they can display also differ. The CIE color space is a set of standardized color representation methods developed by the International Commission on Illumination for accurately describing and comparing colors [22,23]. The CIE color space is a model based on human visual perception, providing a unified and device-independent way to describe colors. The color gamut is a specific manifestation of the color range that can be reproduced by a device or the human eye within the color space [24]. On a color gamut chart, the triangular area enclosed by the three primary colors of red, green, and blue represents the color gamut of the display. The higher the color purity of the three primary colors, the closer their coordinates in the color gamut chart are to the spectral color curve (the boundary of the visible spectrum). This implies that the color range that the display can present is closer to the pure colors in the natural spectrum. When the coordinates of the three primary colors of the display are closer to the spectral color curve, the colors it can display will be more saturated and vivid. Furthermore, the larger the area of the color gamut triangle, the wider the color gamut covered by the display, indicating that the display is capable of presenting more colors. Therefore, the larger the color gamut, the richer the color performance of the display, and the more details and color gradations it can reproduce. The size of the color gamut of a display directly affects the accuracy and richness of its colors. Wide-color-gamut displays are typically more significant in applications such as image processing, professional design, and high-definition television because they can restore various colors more accurately and richly [23,25,26,27,28].

FWHM represents the width of the emission spectral line. Specifically, it is the distance between the two points on the emission intensity curve at half the peak intensity (half-peak). In the spectrum, the difference between the wavelength values corresponding to these two points is the FWHM, which can be used to quantify the spectral width or color purity of the luminescent material [29].

In display technology, multiple light sources with narrow FWHM (such as red light, green light, and blue light) are typically used to cover the entire color gamut. The FWHM of each light source is narrow, so the representation of each color is more precise and saturated. When combining these narrow FWHM light sources, their color gamuts will cover different areas in the color gamut chart, and these areas are often close and non-overlapping, thereby covering a wider color range. On the color gamut chart, the boundaries of the color gamut are usually defined by the most extreme colors that the display can accurately reproduce. Light sources with narrow FWHM can position the boundaries of the color gamut more precisely. For example, for red, green, and blue, the narrower the FWHM of each light source, the more accurately the boundaries of the color gamut can be restored, thereby expanding the color range that the display can display. A narrow FWHM enables the display to better restore the details of the spectrum and highly saturated colors without causing color blurring due to a broad color spectrum [30].

The FWHM of quantum dot materials is influenced by several key factors, including material composition and type, size distribution, synthesis methods, surface defects, and ligand modification. Specifically, different materials, such as CdSe, CdTe, InP, PbS, etc., exhibit distinct electronic structures and bandgap characteristics, leading to variations in emission spectral widths among various types of quantum dots. For example, perovskite quantum dots inherently possess a relatively narrow FWHM (<30 nm) [31,32,33,34,35,36,37,38,39,40,41,42,43,44,45], whereas CuInS_2_ exhibits a broader FWHM (>100 nm) [46]. Moreover, a wide size distribution of quantum dots results in significant variations in emission wavelengths, thereby broadening the spectrum and increasing the FWHM. Reducing the size distribution, particularly minimizing its standard deviation, is critical for enhancing spectral quality and narrowing the FWHM. Surface defects and vacancies can also alter the electronic energy levels of quantum dots, causing spectral shifts or broadening. Surface modifications using organic ligands or inorganic shells can improve surface quality, reduce defects, and consequently narrow the emission spectrum [47,48]. Precise control over synthesis conditions, surface modification, and optimization of the reaction environment can effectively minimize the size distribution of quantum dots, resulting in narrower spectra and smaller FWHM, thereby enhancing the performance of quantum dot materials in display, optoelectronic, and other applications [41,45].

### 2.2. Backlight Display Brightness and Quantum Dot Photoluminescence Quantum Yield

The brightness of a backlight display is of paramount significance for display performance, particularly in aspects such as presentation of high dynamic range (HDR) content, contrast, and color manifestation in strong light environments. High brightness not only conspicuously enhances visibility, making the screen content more legible in bright settings, but also boosts the layering and detail presentation of images. It assists the display in presenting more elaborate details, especially in the dark and highlight sections, guaranteeing a fine and realistic picture [49]. Simultaneously, a higher brightness facilitates more precise color reproduction, elevating color saturation and vividness, thereby offering a more vivid and natural visual experience [50].

Photoluminescence quantum yield (PLQY) is a crucial parameter that describes the photoluminescence efficiency of materials. It is defined as the ratio of the number of photons re-emitted through radiative transitions (luminescence) to the number of photons absorbed by the material.

In backlight displays, fluorescent materials are typically employed to convert the light generated by blue or ultraviolet light sources into other colors (such as red and green), thereby achieving a complete color gamut and rich color performance. The higher the PLQY of the fluorescent material, the greater the portion of absorbed light energy that is converted into visible light, consequently enhancing the brightness of the display. Additionally, fluorescent materials with high PLQY typically imply fewer non-radiative losses (such as heat losses) [51]. High losses associated with low PLQY materials might lead to an increase in the temperature of the backlight system, thereby influencing the thermal management and efficiency of the system. With the enhancement of PLQY, not only is the brightness elevated, but the thermal effect is also mitigated, thereby improving the overall energy efficiency and stability of the display system [52,53,54].

### 2.3. Backlight Display Life and Quantum Dot Stability

The lifetime of a backlight display refers to the period during which the backlight source and fluorescent materials can operate normally while maintaining a specific brightness and performance. The lifetime of a backlight display is a critical factor influencing the overall service life, image quality, and user experience of the display. The lifetime of a backlight display is affected by multiple factors, including the quality of the light source and fluorescent materials, the driving current, the operating temperature, the heat dissipation design, the environmental conditions, the brightness setting, the power supply stability, and material aging. Reasonable design and utilization can prolong the lifetime of backlight displays, reduce the phenomenon of light attenuation, and maintain the display effect [55,56].

The stability of quantum dots pertains to the capacity of quantum dots to sustain their physical and chemical properties (such as optical properties, luminescence efficiency, color stability, etc.) over an extended period or under specific conditions. Quantum dots have significant applications in fields such as display technology, optoelectronics, and biological imaging, particularly being widely utilized in quantum dot displays (QLED) and quantum dot backlight displays. Nevertheless, the stability of quantum dots still constitutes a challenge in their commercial applications. High-stability quantum dots are of paramount importance for the persistence of display quality. They are required to maintain long-term brightness and color stability to avoid any impact on display quality due to light attenuation or color variations [57,58].

## 3. Development of Quantum Dot Backlight Display Materials

### 3.1. Group II–VI Quantum Dot Materials

Group II–VI quantum dot materials are semiconductor quantum dots formed by elements of Group II and Group VI in the periodic table. Group II–VI elements encompass elements such as zinc (Zn), cadmium (Cd), mercury (Hg), etc., while Group VI includes elements such as sulfur (S), selenium (Se), and tellurium (Te), etc. Table 1 presents the key parameters of II–VI quantum dot materials reported in recent years. The development of II–VI quantum dot materials in the field of backlight display has undergone a transformation from traditional cadmium-based quantum dots to cadmium-free alternative materials.

**Figure 2:**
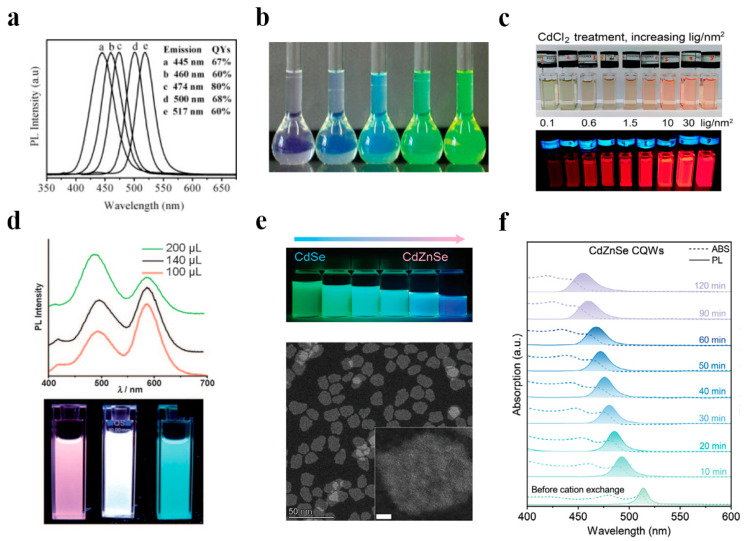
Group II–VI quantum dots. (**a**) PL spectra for CdSe/CdS core–shell nanocrystals with different core size and different shell thickness. (**b**) Photograph of solutions of CdSe/CdS core–shell nanocrystals with different core size and different shell thickness under normal indoor light without UV irradiation. Reprinted with permission from ref. [60]. Copyright 2005 Wiley. (**c**) Photographs under ambient room light (**top**) and UV light (**bottom**) showing effect of CdCl_2_ treatment at increasing CdCl_2_ concentrations on QD PL. Reprinted with permission from ref. [68]. Copyright 2018 American Chemical Society. (**d**) Cu:Mn-ZnSe-doped QD samples with different amounts of Cu precursors. Reprinted with permission from ref. [64]. Copyright 2011 Wiley. (**e**) Solutions of CQWs after different CE reaction times (10 to 60 min) under 365 nm UV light. (**f**) Normalized absorption and PL spectra of CdZnSe CQWs with respect to the CE reaction time. Reprinted with permission from ref. [66]. Copyright 2024 Wiley.

CdSe is the earliest II–VI quantum dot material that has been extensively studied and applied, primarily utilized in quantum dot backlight sources. It possesses a high quantum yield and a broad absorption spectral range (Figure 2a,b), and the emission wavelength can be precisely modulated [59,60]. CdS quantum dots are frequently employed as shell materials to form core–shell structures (such as CdSe/CdS) in conjunction with CdSe cores to enhance light stability and quantum efficiency [60,61]; As illustrated in Figure 2c, due to its relatively smaller bandgap, CdTe is more prevalently utilized in research on red light quantum dots [67,68,69]. These quantum dots are capable of providing high-color-gamut and high-brightness display effects. Nevertheless, the toxicity of cadmium and environmental pollution concerns have elicited apprehensions regarding CdTe usage [70,71,72,73]. The ROHS environmental protection standard of the European Union stipulates that the cadmium content (mass fraction) of quantum dot components should be less than 1 × 10^−4^. From the perspective of the development trend of future quantum dot material technology, low-cadmium and cadmium-free materials will become the options for all end products. To address the environmental and health issues of cadmium-based quantum dots, researchers have actively developed low-cadmium and cadmium-free quantum dot materials [74,75,76,77].

Zinc-based quantum dots are non-toxic and comply with environmental protection requirements, serving as significant candidates for substituting cadmium-based quantum dots. ZnSe and ZnS are the principal alternative materials for cadmium-based quantum dots, and they have emerged as research hotspots for eco-friendly quantum dots due to their non-toxicity and favorable chemical stability: by doping manganese (Mn) or copper (Cu), the luminescent properties of zinc-based quantum dots can be regulated (as shown in Figure 2d), further enhancing their color purity and brightness [62,65]. However, the relatively lower quantum efficiency and brightness of zinc-based quantum dot materials have restricted their further development in the field of backlight display.

Copper indium sulfide (CuInS_2_) quantum dots are a non-toxic and environmentally friendly type of quantum dot that have garnered attention in backlight displays in recent years [46]. They feature a wide optical bandgap and a large absorption coefficient, and the emission color can be adjusted by varying the composition ratio or the size of the quantum dots. Nevertheless, compared with cadmium-based quantum dots, CuInS₂ quantum dots have a broader spectral bandwidth (FWHM > 60 nm), and can only be applied in display applications with lower requirements for color purity. Moreover, the facile degradation of CuInS₂ quantum dots under conditions of high humidity and high temperature also constrains their application in the field of backlight display [78].

Cd_1−x_Zn_x_Se alloy quantum dots strike a balance between the high efficiency of cadmium-based quantum dots and the environmentally friendly nature of zinc-based quantum dots by adjusting the proportion of different elements (Figure 2e,f). However, the homogeneity and composition control of alloy quantum dots pose higher requirements for synthesis techniques, and the relatively high preparation cost of alloy quantum dots limits their application in large-scale production [66].

### 3.2. III–V Quantum Dot Materials

The development of III–V quantum dot materials in the field of backlight display has also made significant advancements, particularly as they typically exhibit non-toxicity and outstanding optical properties, serving as a crucial complement and alternative to II–VI quantum dots (refer to Table 2).

**Figure 3:**
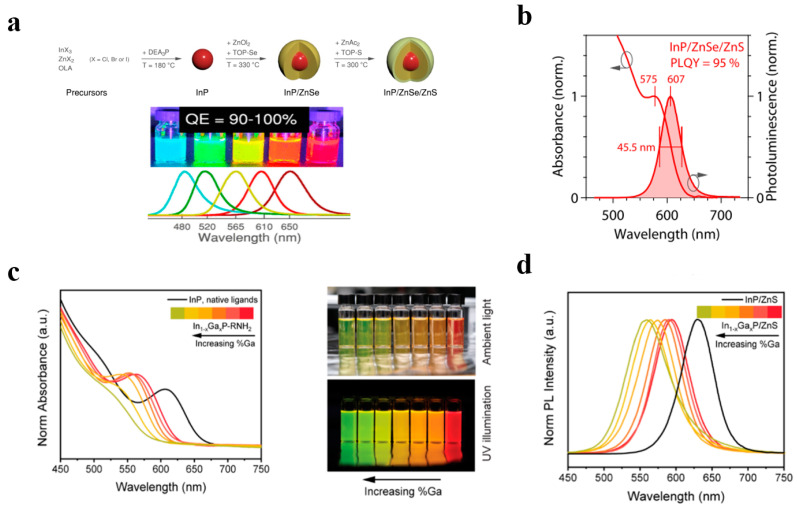
Group III–V quantum dots. (**a**) The synthesis strategy of InP/Zn(Se,S)/ZnS featuring a core–shell–shell structure and the attainment of multiple emission colors through the adjustment of the composition of the inner shell. (**b**) Absorbance and emission spectra of InP/ZnSe/ZnS QDs. Reprinted with permission from ref. [80]. Copyright 2022 American Chemical Society. (**c**) Absorption spectra of alloyed In_1−x_GaxP cores and the large range of emission colors produced by core−shell In_1−x_GaxP/ZnS samples with varying gallium. (**d**) In_1−x_GaxP/ZnS emission spectra. Reprinted with permission from ref. [87]. Copyright 2023 American Chemical Society.

InP quantum dots are among the most focused upon types of III–V quantum dots. Due to their non-toxicity and high performance, they have gradually become an important candidate material for replacing cadmium-based quantum dots. InP quantum dots comply with the RoHS environmental protection requirements, making them highly suitable for consumer-grade backlight displays (Figure 3a,b). In recent years, the quantum efficiency and stability of InP quantum dots have been significantly enhanced through surface passivation and core–shell structure design. Nevertheless, as InP quantum dots tend to generate surface defects, which affect their optical properties and stability, more efficient surface passivation methods are required to suppress the occurrence of surface defects [79,80,81,82].

InAs quantum dots are also a type of direct bandgap semiconductor material, and their emission wavelengths can be achieved by adjusting the size of the quantum dots, covering the full range from the visible-light to the infrared region. In backlight displays, InAs quantum dots are more frequently used in high-end display devices for extending the spectrum (particularly in the deep-red and near-infrared regions). Compared with other quantum dot materials, the preparation process of InAs quantum dots has strict requirements for growth conditions, presenting technical challenges in terms of uniformity and size control [83].

In_1−x_Ga_x_P alloy quantum dots, as environmentally friendly red light materials, have received increasing attention in recent years. Alloyed III–V quantum dots can achieve flexible regulation of the emission wavelength by adjusting the material components (such as the ratios of In, Ga, and P) [85,86]. The emission wavelength range of In_1−x_GaxP quantum dots covers the red band of visible light (as shown in Figure 3c,d), making them extremely suitable for red light compensation in displays: the optimized In_1-x_Ga_x_P quantum dots have high luminous efficiency and have been employed in high-end quantum dot backlight modules. However, compared to common III–V quantum dot materials, the preparation of alloy quantum dots is more complex, and the component control and uniformity of different elements pose higher demands on the synthesis process [87].

### 3.3. Perovskite Quantum Dot Materials

Perovskite quantum dots (PQDs) typically possess an ABX_3_ crystal structure composed of corner-sharing [BX_6_]^4−^ octahedra. The A-site cation is confined within the cubic cage formed by the octahedra, and this distinctive crystal structure enables it to exhibit significant potential in the field of optoelectronics [1].

**Figure 4:**
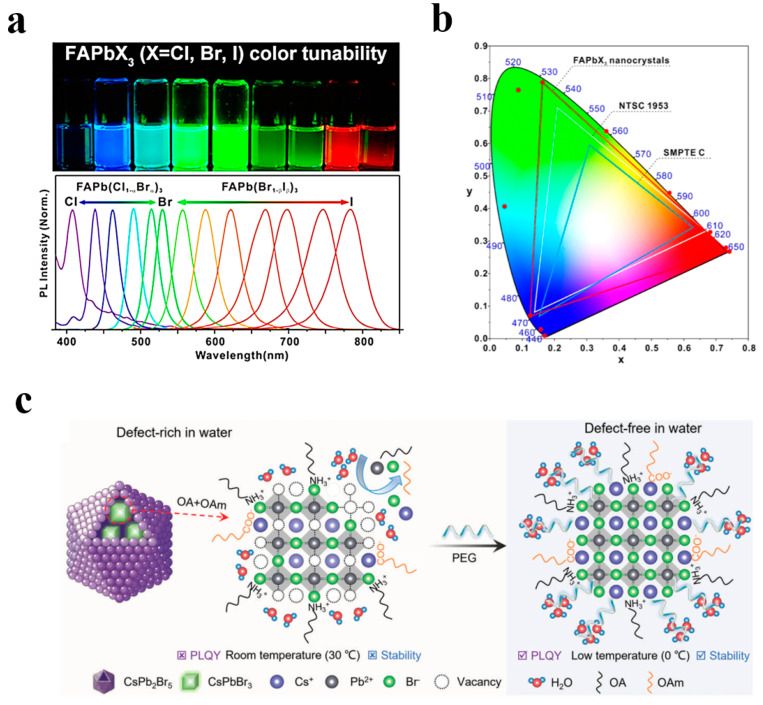
Perovskite quantum dots (**a**) FAPbX_3_ nanocrystals dispersed in toluene under UV irradiation (λpeak = 365 nm) and PL emission spectra of FAPbX_3_ nanocrystals. (**b**) Corresponding color gamut of FAPbX_3_ nanocrystals displayed on the CIE diagram. Reprinted with permission from ref. [93]. Copyright 2017 American Chemical Society. (**c**) Scheme of synthesized aqueous-based CsPbBr_3_/CsPb_2_Br_5_ PQDs using vacancy inhibitors of PEG, forming a defect-free surface in water (OA refers to oleic acid, and OAm represents oleylamine). Reprinted with permission from ref. [91]. Copyright 2023 Wiley.

Common A-site cations include: inorganic cations, such as Cs^+^ and Rb^+^, and organic cations, such as methylammonium (CH_3_NH_3_^+^, abbreviated as MA^+^) and formamidinium cations (CH(NH_2_)_2_)^+^, abbreviated as FA^+^). The selection of the A-site cation has a direct influence on the size matching, tolerance factor, and overall stability of the crystal structure. The B-site ion is located at the center of the octahedron and is typically a divalent metal cation. Pb^2+^ is the most frequently utilized B-site ion, conferring high photoluminescence efficiency and outstanding optoelectronic properties to perovskite [88,89,90,91,92,93]. However, due to the high biotoxicity of Pb^2+^, researchers have been seeking alternative metal ions, such as Sn^2+^, Zn^2+^, and Mn^2+^, etc. These alternative metals can modify the lattice structure or regulate the optical properties, but many metal ions have difficulties in forming an ideal three-dimensional perovskite structure or may lead to bandgap characteristics that are not suitable for specific applications [94]; As depicted in Figure 4a,b, the X-site anion forms [BX_6_]^4−^ octahedra together with the B-site ion and constitutes the core component of the luminescence performance of perovskite materials. Regulation of the emission wavelength can be achieved by varying the halogen type, thereby covering the spectral range from ultraviolet to near-infrared [93].

Table 3 presents the key parameters of perovskite quantum dot materials reported in recent years. PQDs encompass almost all the advantages of luminescent materials, such as high brightness (their photoluminescence quantum yield (PLQY) approaches the theoretical limit), tunable emission (precise wavelength control (from ultraviolet to near-infrared) can be realized by altering the halogen type or doping), high color purity (the emission spectrum is narrow (typical FWHM is 20–40 nm) and the color saturation is extremely high), high light absorption coefficient (the light absorption capacity within the visible light range is extremely strong), high defect tolerance (even if there are defects in the crystal, its optical performance still exhibits excellence), and facile fabrication (mass production can be achieved through low-cost processes such as solution methods, spraying, spin coating, etc. [88,95]).

Although perovskite (LHP) quantum dots have demonstrated tremendous potential and are widely regarded as a star material in the field of backlight applications, their stability issues, especially sensitivity to moisture, oxygen, light, and temperature, still constitute the main impediment restricting their wide application. As shown in Figure 4c, to solve this problem, researchers are enhancing the environmental stability of perovskite quantum dots through methods such as surface modification, optimization of the synthesis process, and encapsulation techniques [89,90,91,92]. The structural flexibility and outstanding optoelectronic properties of perovskite quantum dots have drawn significant attention in the fields of LED and backlight display technologies. With further improvements in stability and environmental performance, their potential in the future commercialization domain will be broader.

## 4. Synthesis of Quantum Dot–Polymer Materials and Their Application in Backlight Display Field

The application of quantum dot–polymer materials in the field of backlight displays is rapidly emerging as a highly focused research hotspot. This type of material ingeniously combines the outstanding optical characteristics of QDs with the processing convenience of polymers, demonstrating immense potential, particularly in enhancing display performance, reducing production costs, and realizing flexible displays [107].

**Figure 5:**
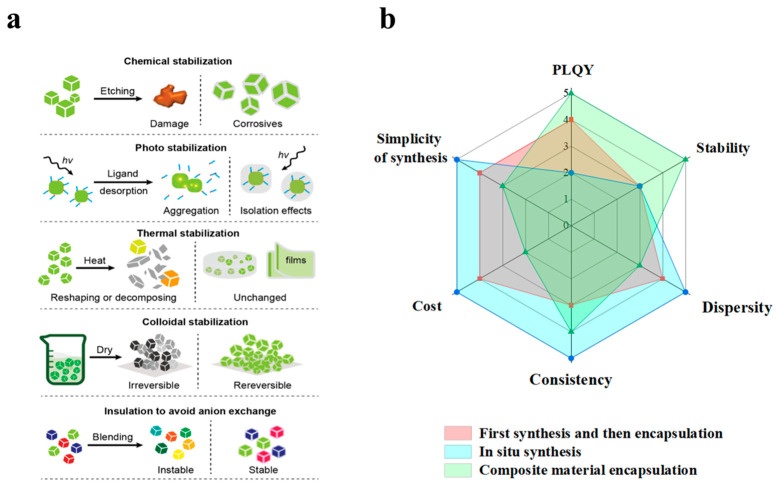
(**a**) Schematic diagram of functions by encapsulation illustrated by CsPbX_3_ QDs. Reprinted with permission from ref. [20]. Copyright 2019 Wiley. (**b**) Radar map of the advantages and disadvantages of three methods of combining QDs with polymers.

Table 4 presents advancements in research on quantum dot–polymer materials in the field of backlight displays in recent years. When quantum dots are combined with polymers, the advantages of both can be fully exploited. Quantum dots offer excellent optical properties, while polymers enhance the processing flexibility, mechanical strength, and environmental adaptability of the materials. As depicted in Figure 5a, the polymer substrate can effectively enwrap the quantum dots, protecting them from environmental factors such as moisture and oxygen, thereby significantly enhancing the stability and durability of the quantum dots [108,109,110]. Additionally, polymers can optimize the dispersion of quantum dots within the material, ensuring their homogeneous distribution, thereby avoiding aggregation and agglomeration phenomena and maintaining the efficient luminescence performance of the quantum dots [111].

As depicted in Figure 5a, through the combination of the properties of quantum dots and polymers, quantum dot–polymer materials can not only enhance the color gamut, brightness, and stability of backlight displays but also reduce production costs, facilitating large-scale manufacturing. With the in-depth progress of related research, quantum dot–polymer materials are anticipated to play a significant role in future display technologies and become an essential material foundation for next-generation displays, lighting equipment, and optoelectronic devices [112,113]. Currently, the common preparation methods of quantum dot polymers include pre-synthesis of quantum dots followed by encapsulation, in situ synthesis of quantum dots in polymers, and encapsulation of quantum dot composite materials in polymers. The pros and cons of the three methods are illustrated in Figure 5b. Hereinafter, we will conduct a detailed discussion on each synthesis method.

### 4.1. Quantum Dots First Synthesized and Then Packaged

Synthesis followed by purification and then encapsulation of quantum dots is currently the most common synthesis approach for quantum dot–polymer materials. The advantage of this method lies in the relatively independent processes of quantum dot synthesis, purification, and polymer encapsulation, which can guarantee the high quality and stability of quantum dots, thereby enhancing the overall performance of the polymer composite materials. Nevertheless, although this process can achieve high purity and good luminescent performance of quantum dots, it still confronts several challenges. For instance, the synthesis, purification, and encapsulation processes are complex, requiring multiple steps. There exist interface issues between the polymer and quantum dots with poor compatibility. Quantum dots are prone to aggregation during the encapsulation process. Moreover, during the encapsulation, the polymer matrix material may not completely cover the quantum dots or the encapsulation may be incomplete, resulting in the exposure of quantum dots to the external environment and subsequently reducing their stability.

**Figure 6:**
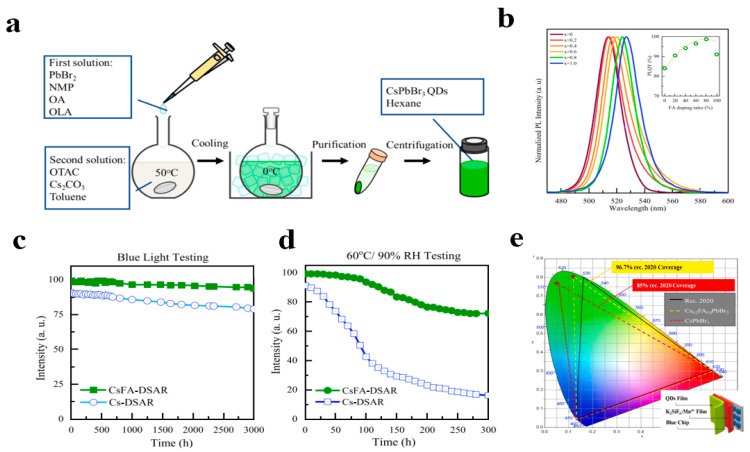
(**a**) Flowchart of the dual-solvent assisted reprecipitation (DSAR) technique. (**b**) Photoluminescence spectra (the inset shows photoluminescence quantum yield). (**c**) Blue light stability test. (**d**) The 60 °C/90%RH stability test. (**e**) Color gamut of the fabricated devices using the Cs-DSAR and CsFA-DSAR PQDs. Reprinted with permission from ref. [38]. Copyright 2023 Elsevier.

As depicted in Figure 6a–e, Lu et al. synthesized high-quality Cs_1−x_FA_x_PbBr_3_ quantum dots using a dual-solvent-assisted reprecipitation method [38]. Subsequently, the purified quantum dots were mixed with MMA to fabricate quantum dot–polymer films. The Cs_0.2_FA_0.8_PbBr_3_ quantum dot–polymer film maintained 98% of its initial strength under normal temperature and humidity conditions. After being exposed to 60 °C/90% relative humidity for 300 h, it retained 75% of its initial strength, demonstrating outstanding environmental stability. The white light-emitting device fabricated using the synthesized PQD, K_2_SiF_6_:Mn^4+^ phosphor, and blue LED chip achieved a Rec. 2020 color gamut coverage rate of 96.7%. The quantum dot–polymer material synthesized by this method possesses high efficiency, environmental reliability, and a wide NTSC 2020 coverage range, and is regarded as a promising candidate in display devices.

### 4.2. Quantum Dots Synthesized In Situ in Polymers

In situ synthesis enables the direct generation of quantum dots within the polymer matrix, ensuring their homogeneous distribution within the polymer material. This circumvents the issues of quantum dot agglomeration or uneven dispersion that might arise in conventional approaches, guaranteeing the consistency and stability of the material’s optical and mechanical properties.

Unlike the pre-synthesis of quantum dots using solution or solid-state methods followed by polymer encapsulation, in situ synthesis permits precise control over the growth process of quantum dots during the polymer synthesis, achieving more accurate size control and a more uniform distribution, while maximizing the retention of their outstanding optical characteristics. Compared to physical mixing methods, in situ synthesis enhances the interaction between quantum dots and the polymer through a chemical synthesis process, reducing interfacial defects or stress concentration, thereby enhancing the stability of the composite material [114].

During in situ synthesis, the interface between the quantum dots and the polymer is formed naturally through chemical reactions and intermolecular interactions, rendering the compatibility between the quantum dots and the polymer matrix more intimate. This direct binding contributes to improving the interface contact between the two, thereby enhancing the photoelectric performance of the quantum dots and strengthening the mechanical properties of the polymer material. Moreover, in situ synthesis enables the direct embedding of quantum dots during the synthesis of the polymer matrix, eliminating the need for additional post-treatment steps, reducing the complexity and cost of the manufacturing process. This is particularly significant for large-scale production, enabling low-cost and straightforward production procedures. Additionally, in situ synthesis obviates the requirement for additional dispersants or surface modifiers, avoiding potential chemical contamination or instability and enhancing the simplicity and controllability of the process.

**Figure 7:**
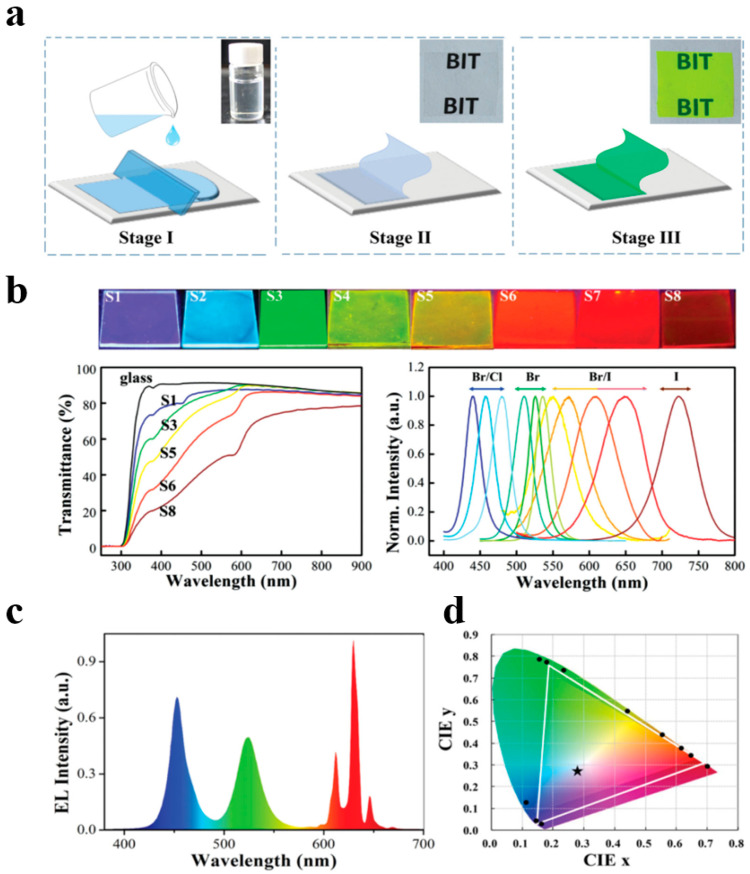
(**a**) Schematic illustration of the in situ fabrication of MAPbBr_3_ NCs-embedded PVDF composite films. (**b**) Optical images under a UV lamp (365 nm) of color-tunable MAPbX_3_–PVDF composite films with different halogen constitutions on glass substrates. (**c**) Emission spectrum of the white LED using green emissive MAPbBr_3_–PVDF composite films and red emissive phosphor. (**d**) The color coordinate (star) and the white triangle (white line) of obtained white LED in CIE 1931 diagram. Reprinted with permission from ref. [32]. Copyright 2016 Wiley.

As illustrated in Figure 7a–d, Zhang et al. dissolved MAX, PbX_2_ (x = Cl, Br, I), and PVDF simultaneously in DMF to prepare a precursor solution. Subsequently, they utilized a reduced-pressure environment to rapidly remove the solvent and in situ-synthesized MAPbX3–PVDF polymer films [32]. The interaction between the -CF_2_- groups in PVDF and the organic A-site MA^+^ led to uniform size and spatial distribution of the fabricated quantum dots in the composite films. These quantum dot–polymer films exhibited superior PL performance, with a PLQY as high as 94.6 ± 1%. The combination of green-emitting quantum dot–polymer films with red-emitting K_2_SiF_6_:Mn^4+^ resulted in a backlight display device with high luminous efficiency (up to 109 Im W^−1^ at 20 mA) and a wide color gamut (121% of the NTSC standard), providing possibilities for quantum dot–polymer LCD backlight displays.

### 4.3. Quantum Dots Encapsulated In Polymers

Quantum dot composite materials refer to composites formed by combining quantum dots with other materials (such as glass, metals, inorganic materials, etc.). By integrating the excellent optical properties of quantum dots with the characteristics of the matrix materials, quantum dot composite materials can exhibit distinctive advantages in multiple fields. Currently, common quantum dot composite materials include quantum dot–glass composites and quantum dot core–shell composites.

**Figure 8:**
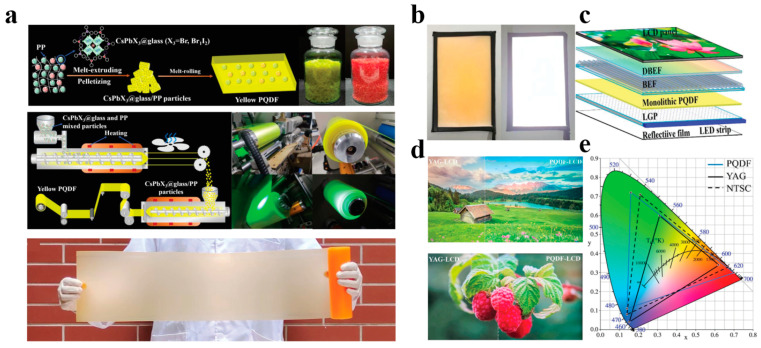
(**a**) Schematic diagrams of the preparation procedure for yellow PQDF via melt extruding-rolling method. Photographs of the as-prepared PP-encapsulated CsPbX_3_@glass composite particles and the corresponding green and yellow PQDFs. (**b**) Photograph of yellow monolithic PQDF-based backlit unit and luminescent image of the backlit unit at an operating voltage of 12 V. (**c**) Schematic structure of an LCD prototype using yellow PQDF as a light converter. (**d**) Comparison of the display performance of a YAG-based LCD and PQDF-based LCD. (**e**) Color gamut of PQDF-based LCD (blue solid triangle), NSTC 1953 standard (black dashed triangle), and YAG-based commercial LCD (black solid triangle). Reprinted with permission from ref. [37]. Copyright 2024 Wiley.

Quantum dot–glass composites are formed by growing quantum dots in situ at high temperatures in glass, confining the quantum dots within the dense glass network structure to restrict their size. Additionally, the excellent waterproof performance of glass can significantly enhance the thermal and water stability of quantum dots. As depicted in Figure 8, Lin et al. first mixed quantum dot raw materials and borosilicate glass components, and synthesized quantum dot–glass composites using the melt-quenching method followed by annealing treatment. Subsequently, the quantum dot–glass composites were ground into powder and fabricated into a large-area yellow single-layer quantum dot–polymer film encapsulated with PP through industrial melting extrusion and rolling methods [37]. The photoluminescence quantum yield (PLQY) of the quantum dot–polymer film was as high as 92%, with a narrow FWHM of 19 nm (green) and 33 nm (red). Significantly, the quantum dot plate could pass the stringent aging test at 85 °C/85% RH and achieve a working T90 lifetime of over 1000 h. Finally, by coupling the yellow single-layer quantum dot–polymer film with a blue light guide plate, a white backlight unit was designed. The constructed prototype display possesses superior color rendering performance, with narrow green/red emissions and a color gamut reaching 110% of the National Television System Committee (NTSC).

Early researchers discovered that defects on the surface of quantum dots, particularly surface vacancies and unsaturated bonds, frequently led to the occurrence of non-radiative recombination processes, which significantly reduced the luminescence efficiency and optical stability of quantum dots [114,115,116,117,118]. These defects not only result in the energy loss of light but also may cause the photodegradation of quantum dots, severely influencing their luminescence performance during long-term usage, especially in environmental conditions such as variations in humidity and temperature. Hence, how to effectively suppress the surface defects of quantum dots and enhance their luminescence efficiency and stability has emerged as a significant challenge for researchers [47,48].

To address this issue, quantum dot core–shell composite materials have emerged. By coating the surface of quantum dots with a shell layer possessing excellent properties (typically semiconductor or metal materials), researchers can effectively seal or repair the surface defects and reduce the occurrence of non-radiative recombination, thereby significantly enhancing the fluorescence quantum efficiency, optical stability, and environmental adaptability of quantum dots. The core–shell structure not only protects the core of quantum dots from the external environment but also further improves their optical characteristics, such as tunable emission wavelengths, high brightness, and longer photodegradation time, through the rational design of the combination of core and shell materials [117,118]. Therefore, quantum dot core–shell composite materials exhibit extensive application prospects in fields such as display technology, optoelectronic devices, sensors, biomedicine, etc., and have become one of the effective approaches to address the issue of optical performance degradation of quantum dots.

**Figure 9:**
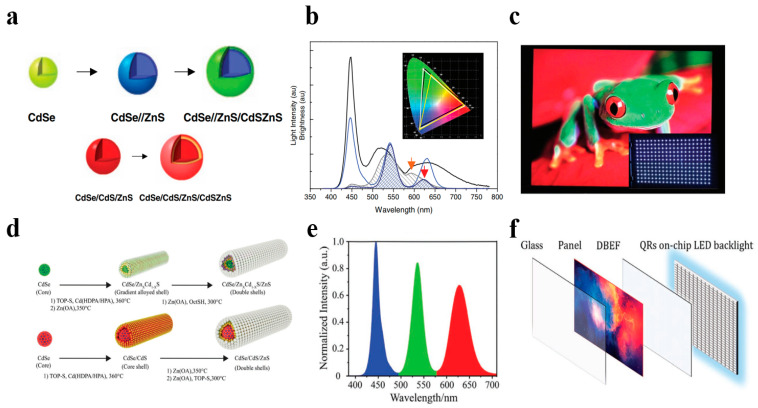
(**a**) Schematic structures of the growth of green and red QDs. (**b**) Light intensity spectra (solid line) and brightness (hatched area) of QD-LED (blue) and phosphor-LED (gray). Inset: color triangles of QD-LED (white) and phosphor-LED (yellow) compared to NTSC1931 (black). (**c**) Display image of a 46-inch LCD TV panel and a quarter of the white QD-LED backlights (inset). Reprinted with permission from ref. [43]. Copyright 2010 Wiley. (**d**) Schematic of syntheses of the ZnS modified green and red QRs. (**e**) The spectrum of the fabricated QRWLED consists of three emission band peaks at 450, 527, and 624 nm. (**f**) Schematic of a QRs on-chip backlight with dual-brightness-enhancement film (DBEF) design for displays. Reprinted with permission from ref. [44]. Copyright 2021 Wiley.

As depicted in Figure 9, Jang et al. synthesized green-emitting CdSe/ZnS/CdSZnS and red-emitting CdSe/CdS/ZnS/CdSZnS multilayer core–shell quantum dot composite materials using the thermal injection method and subsequently mixed them with organosilicon to prepare quantum dot–polymer films [43]. Due to the passivation of the quantum dot surface by the multi-core–shell structure, the luminescence efficiency of these quantum dots reached 100%. The quantum dots were encapsulated in blue LEDs and used as green and red color converters to fabricate white LEDs for backlight display. The equivalent quantum effects of green and red QD-LEDs reached 72% and 34%, respectively, and the QD-LEDs maintained their initial efficiency for over 2200 h in a normal working environment. The white QD-LED, adjusted to (0.24, 0.21) in CIE 1931 for backlight applications, had a luminous efficiency of 41 lm/W, and the color reproducibility was 100% compared to the NTSC color space. Moreover, the white QD-LED backlight was successfully integrated onto a 46-inch liquid crystal television panel, demonstrating excellent color gamut.

As shown in Figure 9, Kang et al. synthesized rod-shaped CdSe/Zn_x_Cd_1−x_S/ZnS and CdSe/CdS/ZnS multi-core–shell structure quantum dot materials with a 1D structure using the thermal injection method, addressing the PLQY quenching and stability issues of rod-shaped quantum dot materials [44]. The introduction of the outer gradient Zn_x_Cd_1−x_S shell layer and ZnS shell layer in the CdSe/Zn_x_Cd_1−x_S/ZnS gradient alloy quantum rods provided a smoother confinement potential, reduced non-radiative energy transfer, and achieved a solid-state PLQY of 81%. The white light quantum rod polymer prepared by mixing with silica gel achieved an astonishing luminous efficiency of 149 lmW-1, with a color gamut of 118% NTSC and 90% BT2020, possessing high thermal stability and optical stability and being highly suitable for LCD backlight applications. Additionally, it showed higher energy efficiency compared to the most advanced LED backlighting devices, with a maximum efficiency of an astonishing 200 lm/W. Although the efficiency decreased to 120 lm/W at higher currents (50 mA), this is still much higher than that of phosphor-based LEDs (50 lm/W).

## 5. Conclusions and Prospects

Quantum dot–polymer materials (QDPs) have exhibited tremendous application potential in the field of backlight display, especially in enhancing color performance, brightness, efficiency of displays, and reducing costs. Quantum dot materials, with their outstanding optical properties such as high luminance, tunable emission wavelengths, and high color purity, have offered a novel impetus for the innovation of backlight display technology. Polymer materials in turn, due to their favorable processability, tunability, flexibility, and advantages in large-scale production, have addressed the stability and processing difficulties that quantum dot materials might encounter in practical applications. By combining quantum dots and polymers, it is possible to maintain high optical performance while endowing the materials with better processability and durability.

At present, significant advancements have been achieved in the application of quantum dot–polymer materials in backlight display, particularly in quantum dot-enhanced liquid crystal displays (QLED) and quantum dot backlight units (QBLUs), which can offer a broader color gamut, higher luminance, and longer service life [119,120,121,122]. Through optimizing the size, composition, and surface modification of quantum dots, as well as the structure and performance of polymer substrates, researchers have continuously enhanced the performance of these materials, making their application in high-end display devices more mature and widespread [123,124].

In the future, research on quantum dot–polymer materials in the field of backlight display will evolve towards more efficient, stable, and economical directions. Specifically, the following areas will be the key points of future research.

(1) Material stability and environmental adaptability: Despite the fact that quantum dot–polymer composite materials have achieved favorable results in laboratories, in practical applications, environmental factors such as temperature, humidity, and ultraviolet radiation remain critical in influencing their long-term stability. Hence, in the future, more materials with high environmental stability need to be developed, and their durability can be further enhanced through innovative surface modification or encapsulation techniques.
(2) High-efficiency light conversion technology: Although quantum dots themselves possess excellent optical properties, how to enhance the photoelectric conversion efficiency of quantum dots and reduce energy loss remains a crucial aspect for future development. Exploring new quantum dot materials, such as perovskite quantum dots and two-dimensional quantum dots, might provide new breakthroughs for high-efficiency display materials.
(3) Flexible display technology: With the rise of flexible display technology, the application of quantum dot–polymer materials in bendable and stretchable display devices holds broad prospects. The combination of the flexibility of polymers and the optical characteristics of quantum dots can bring about a brand-new display experience. Therefore, future research needs to explore more quantum dot–polymer composite materials suitable for flexible substrates while ensuring that their performance is not compromised during the flexibilization process.
(4) Low-cost, large-scale production: Currently, the production cost of quantum dot–polymer materials is relatively high, restricting their large-scale application in the consumer electronics field. Future research will focus on developing low-cost and straightforward preparation methods, such as solution methods and large-area coating techniques, to meet the requirements of large-scale production and commercialization.

In conclusion, quantum dot–polymer materials have broad application prospects and significant market potential in the field of backlight display. With the continuous optimization of material performance and the advancement of production technologies, they will play a vital role in a wider range of display technologies in the future, contributing to the realization of more efficient, higher-quality, and more innovative display devices.

## Figures and Tables

**Table 1:** Group II–VI quantum dot materials.

Materials	Emission Peak (nm)	FWHM (nm)	PLQY (%)	Ref.
CdSe	550	150	90	[59]
CdSe/CdS	470–570	30	60–80	[60]
CdSe/CdS/ZnS	620	/	75	[61]
ZnSe:Mn	580	50	90	[62]
ZnSe:Cu	508	18	/	[63]
ZnSe:Mn-Cu	490/585	/	13–17	[64]
(Zn,Se)Te/ZnSe/ZnS	463	63	95	[65]
CuInS_2_/ZnS	920	217	65	[46]
(Cd,Zn)Se/ZnS	455–512	<20	81	[66]
CdTe/CdS/ZnS	561	47	73	[67]
CdTe:In	635	/	90	[68]
CdTe:Mn	496–542	<50	/	[69]

**Table 2:** Group III–V quantum dot materials.

Materials	Emission Peak (nm)	FWHM (nm)	PLQY (%)	Ref.
InP/Zn(Se,S)/ZnS	510	45	95	[79]
InP/Zn(Se,S)/ZnS	480–530	45	>90	[80]
InP/ZnSe/ZnS	535	35	90	[81]
InP/ZnS/ZnS	468	47	45	[82]
InP	650	/	24	[83]
InAs	700	/	11	[83]
GaP	400–520	75	35–40	[84]
In_1−x_Ga_x_P/ZnS	490–640	50	46	[85]
In_1−x_Ga_x_As/ZnS	860	/	9.8	[85]
In_1−x_Ga_x_P/ZnS	486	46	65	[86]
In_1−x_Ga_x_P/ZnS	550–630	60	80	[87]

**Table 3:** Perovskite quantum dot materials.

Materials	Emission Peak (nm)	FWHM (nm)	PLQY (%)	Ref.
CsPbCl_3_:Y	404	/	60	[96]
CsPbCl_3_:Cd	381–410	/	60.5	[97]
CsPbCl_3_	405	10.6	71	[98]
CsPbCl_3_:Ni	408	/	96.5	[99]
CsPbCl_3_:Mn	585	/	27	[100]
CsPbCl_3_:Mn	579	80	54	[101]
CsPbBr_3_	522	18	71.3	[102]
CsPbBr_3_	517	18	94.6	[88]
CsPbBr_3_@PbSO_4_	522	16	99.8	[89]
CsPbBr_3_@Cs_4_PbBr_6_	515	20	92	[90]
CsPbBr_3_@CsPb_2_Br_5_	518	16	96	[91]
MAPbBr_3_@PbBr(OH)	514	28	71.5	[92]
FAPbBr_3_	530	22	85	[93]
CsPbI_3_	640	/	88.2	[88]
CsPbI_3_	660	/	88.1	[91]
CsPb(Br_0.4_, I_0.6_)_3_	641	32	32.4	[103]
CsPbI_3_	690	31	100	[104]
FA_0.1_Cs_0.9_PbI_3_	690	45	>70	[105]
FAPbI_3_: Sr	735	/	100	[106]

**Table 4:** Quantum dot–polymer materials for backlight displays.

QDs	Polymer	NTSC1953	PLQY	Light Stability	Ref.
CsPbX_3_	PVDF	107%	/	>80 h	[31]
MAPbBr_3_	PVDF	121%	94.6%	>400 h	[32]
CsPbX_3_	PVDF	128%	70%	50 Day	[33]
FAPbBr_3_	PVDF	118%	99%	>7 Day	[34]
CsPbBrI_2_/Glass	PS	125%	36.9%	40 Day	[35]
(Zn,Cd)(Se,S)/ZnS	PP	120%	>75%	/	[36]
CsPbX_3_/Glass	PP	110%	>90%	>1000 h	[37]
Cs_1−x_FA_x_PbBr_3_	PMMA	/	99%	>90 Day	[38]
CsPbBr_3_/SDDA	PMMA	102%	63%	/	[39]
CsPb(Br/I)_3_/Glass	PMMA	120%	53%	>100 h	[40]
Cs_4_PbBr_6_/CsPbBr_3_	PMMA	131%	85%	/	[41]
CdSe/CdS & (Cd,Zn)(Se,S)/ZnS	PDMS	133%	98%/97%	800 h	[42]
CdSe/ZnS/CdSZnS & CdSe/CdS/ZnS/(Cd,Zn)S	Silicone Encapsulant	100%	100%	>2200 h	[43]
CdSe/Zn_x_Cd_1−x_S/ZnS & CdSe/CdS/ZnS	Silicone glue	118%	81%	>150 h	[44]
CsPbBr_3_/Glass	Silicone glue	126%	86%	/	[45]
